# IA-3DGS Identity-Anchored Dynamic Gaussian Splatting for Long-Term Facial Detail Preservation

**DOI:** 10.3390/s26154947

**Published:** 2026-08-05

**Authors:** Ze Zhao, Lili Yin, Shuaijie Wang, Jie Gao

**Affiliations:** School of Computer Science and Technology, Harbin University of Science and Technology, Harbin 150080, China; 2420410178@stu.hrbust.edu.cn (Z.Z.); 2420410175@stu.hrbust.edu.cn (S.W.); 2420410126@stu.hrbust.edu.cn (J.G.)

**Keywords:** 3D Gaussian splatting, dynamic head avatar, monocular video, identity preservation, Jacobian regularization, long-sequence facial animation

## Abstract

Dynamic 3D Gaussian Splatting (3DGS) enables efficient facial-avatar rendering but may suffer from cumulative geometric drift and degradation of identity-specific details when applied to long monocular sequences. To address this problem, we propose IA-3DGS, an identity-anchored dynamic Gaussian framework for long-term facial animation. The proposed framework contains three main components. First, a 3D morphable model-guided semantic initialization strategy associates Gaussian primitives with anatomically meaningful facial regions. Second, a region-weighted Jacobian rigidity regularizer suppresses non-physical shear and anisotropic stretching in quasi-rigid facial regions while preserving sufficient flexibility in expression-sensitive regions. Third, a cyclic memory correction mechanism periodically aligns the current identity representation with a frozen reference representation and applies exponential moving average smoothing to reduce correction-induced temporal discontinuities. Experiments were conducted on the NeRSemble and NHA datasets and on a self-collected Custom-5K dataset containing continuous monocular facial sequences longer than 5000 frames. IA-3DGS achieved a PSNR of 31.85 dB, an SSIM of 0.942, and an LPIPS of 0.038 on standard-length sequences. At frame 5000, the method obtained an L-LPIPS of 0.046 and a landmark mean error of 1.3 mm, compared with 0.245 and 6.8 mm, respectively, for the purely implicit dynamic 3DGS baseline. The system rendered 1920 × 1080 images at approximately 48 frames per second on a single NVIDIA RTX 4090. These results indicate that the proposed semantic, geometric, and temporal constraints improve long-sequence identity consistency under the evaluated subject-specific monocular setting.

## 1. Introduction

Creating highly realistic and animatable 3D digital humans is a central challenge in the fields of computer graphics and computer vision, driven primarily by cutting-edge applications such as the metaverse, remote interaction, and virtual live streaming [1]. Although Neural Radiance Fields (NeRF) have achieved unprecedented quality in new-view synthesis, the enormous computational overhead of their volumetric rendering remains a major obstacle to real-time interaction [2]. Recently, 3D Gaussian Splatting (3DGS) has emerged as an efficient alternative. By using explicit anisotropic Gaussian primitives and tile-based rasterization, it supports photorealistic rendering at real-time frame rates [3]. Consequently, researchers have rapidly extended 3DGS to the realm of dynamic scenes (Dynamic 3DGS) by coupling Gaussian primitives in canonical space with deformation fields to model physical motion [4]. Despite these advances, one key challenge remains unresolved: the long-term stability of dynamic 3DGS. In real-world deployment scenarios (such as continuously operating AI customer service agents or virtual anchors), digital avatars must maintain strict identity consistency across thousands of consecutive frames. However, current dynamic 3DGS frameworks primarily rely on implicit multilayer perceptrons (MLPs) to predict frame-by-frame spatial offsets. Due to the lack of explicit topological memory, these “black-box” deformation fields struggle to maintain the constancy of high-dimensional features during long-term recursive inference. As a result, minute prediction errors accumulate across consecutive frames, leading to “identity drift” and “texture over-smoothing”; that is, the gradual blurring or geometric distortion of unique high-frequency facial fingerprints such as the corners of the eyes and lip lines [5]. To address the severe challenges of temporal incoherence and error accumulation, this paper proposes Identity-Anchored Dynamic 3DGS (IA-3DGS), a unified framework designed to impose explicit semantic and physical constraints on dynamic deformation processes. We hypothesize that structural collapse in long-term 3DGS does not stem from a deficiency in the expressive power of 3DGS itself, but rather from a lack of explicit anchoring of high-dimensional semantics and temporal memory correction during the optimization process. Specifically, we use a 3D morphable model (3DMM) to initialize anatomically anchored and densely sampled surface primitives, thereby associating the originally unordered Gaussian primitives with facial structures that have explicit anatomical correspondence [6]. Furthermore, we introduce a region-weighted Jacobian rigidity regularizer to encourage locally near-isometric deformation in anatomically stable facial regions, thereby reducing non-physical shear and anisotropic stretching [7]. Finally, we integrate a cyclic memory correction mechanism to dynamically calibrate the current identity state and continuously suppress error accumulation in long sequences. The main contributions of this study are summarized as follows.

(1) We introduce a 3DMM-guided semantic initialization strategy that establishes persistent anatomical correspondence between Gaussian primitives and facial regions in canonical space.
(2) We formulate a region-weighted Jacobian rigidity regularizer. Unlike a uniform rigid-body constraint, the proposed formulation applies stronger regularization to quasi-rigid facial regions and weaker regularization to expression-sensitive regions.
(3) We introduce a cyclic memory correction mechanism that periodically measures identity deviation from a frozen reference representation and uses exponential moving average smoothing to reduce correction-induced temporal discontinuities.
(4) We evaluate IA-3DGS on public facial datasets and on continuous monocular sequences longer than 5000 frames using reconstruction, perceptual, geometric, temporal, and cumulative controlled component analyses.

**Figure 1:**
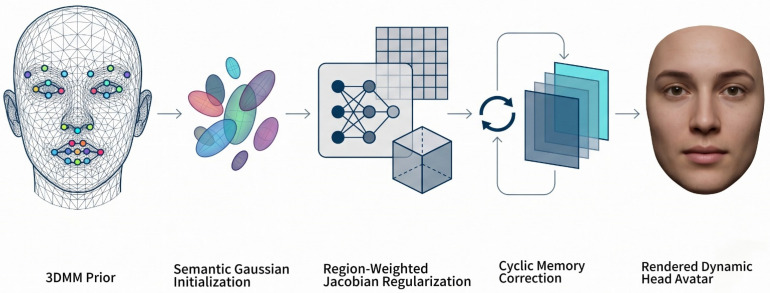
Overview of the proposed IA-3DGS framework. A 3DMM-guided semantic initialization module establishes anatomical correspondences in canonical space. The deformation network predicts expression-dependent non-rigid offsets, the region-weighted Jacobian term regularizes local deformation, and the cyclic memory correction module reduces accumulated identity drift.

The overall architecture of IA-3DGS is illustrated in Figure 1.

## 2. Related Work

### 2.1. 3D Neural Reconstruction and Rendering of Dynamic Scenes

High-fidelity reconstruction and new-view synthesis of dynamic scenes have long been central challenges in the field of computer vision [8]. Early Neural Radiance Fields (NeRF) achieved highly realistic lighting and geometric modeling in static scenes through implicit neural representations [2]. Subsequently, researchers introduced the temporal dimension, leading to the development of dynamic radiance field methods such as D-NeRF and HyperNeRF, which handle dynamic non-rigid deformations by learning deformation mappings from the observation space to the canonical space [9]. However, NeRF and its dynamic variants rely heavily on dense volumetric ray sampling, resulting in extremely high computational costs that make it difficult to meet the urgent demand for real-time interaction in applications such as digital humans [10]. The introduction of 3D Gaussian Splatting (3DGS) technology substantially improved this performance bottleneck. By utilizing anisotropic Gaussian primitives for explicit spatial representation and combining them with tiled differentiable rasterization techniques, it achieved real-time rendering frame rates far exceeding those of NeRF [3]. Subsequently, methods such as Dynamic 3DGS and Deformable 3DGS rapidly extended 3DGS to dynamic scenes. Their mainstream paradigm involves maintaining a Gaussian point cloud in a canonical space and using a multilayer perceptron (MLP) to predict the displacement and deformation for each frame [4,11]. However, as pointed out in this paper, when processing long-duration animations (e.g., exceeding 3000 frames), such deformation networks based on purely implicit MLPs are highly prone to frame-by-frame accumulation of prediction errors due to the lack of globally consistent physical topological constraints, which in turn leads to the gradual collapse of scene structure and the loss of high-frequency details [5,12].

### 2.2. High-Fidelity Avatar Synthesis of the Head and Face

Regarding the 3D reconstruction of digital human heads, early work largely relied on mesh-based deformation transfer; however, this method struggles to handle complex, non-fixed topological structures such as hair and the interior of the mouth [13]. With the rise of neural rendering, methods such as INSTA have incorporated 3D morphable model parameters into the radiance-field representation, enabling pose-driven head avatar synthesis [14]. However, due to the rendering speed limitations of NeRF, such methods are difficult to deploy at scale [15]. Recently, Gaussian-based avatar methods, including GaussianAvatars, GauHuman, HUGS, SplattingAvatar, and 3DGS-Avatar, have integrated facial pose and expression parameters with Gaussian primitives to support high-quality avatar rendering [4,5,16,17,18]. However, most of the aforementioned methods have been evaluated in controlled multi-view environments or on short video clips. When directly applied to monocular video and driven by a long time series, the inherent depth ambiguity in monocular vision, coupled with the models’ lack of explicit, continuous tracking capabilities for subtle facial features (such as crow’s feet and lip lines), inevitably leads to “identity drift” and “over-smoothing” [5,19]. In contrast, the IA-3DGS proposed in this paper is specifically designed for monocular long-time-series-driven tasks and addresses the problem of identity conservation in dynamic 3DGS over long time spans through semantic anchoring techniques [6].

### 2.3. 3D Facial Priors and Physical Rigidity Constraints

To improve the robustness of 3D reconstruction, the current standard approach is to introduce structural priors and physical constraints with strong generalization capabilities [20]. In facial reconstruction tasks, 3DMM and its variants (such as FLAME) provide excellent anatomical topological priors [21]. Some recent Gaussian-avatar methods, such as FastAvatar, use parametric facial priors to initialize or control Gaussian primitives [6]. PointAvatar represents deformable head avatars using point-based primitives and learned deformation fields [22]. However, initialization alone does not explicitly constrain cumulative deformation during long-sequence inference. On the other hand, to prevent non-physical deformations of non-rigid objects, several dynamic reconstruction methods have introduced geometry-aware surface constraints to improve local deformation stability [23,24]. However, these constraints primarily target the relative distances between adjacent Gaussian points and cannot effectively prevent the gradual drift of global structure or the tearing of the deformation field itself. This paper directly introduces a Jacobian rigidity constraint into the implicit deformation field, mathematically enforcing orthogonality in local transformations of the deformation field network. Combined with explicit semantic anchors based on 3DMM, we construct a dual-structure safeguard, thereby ensuring that the digital human maintains physically meaningful facial geometric authenticity and consistency even after thousands of frames of complex expression-driven facial sequences [7,24]. Relationship to existing methods: Existing avatar-reconstruction methods use parametric facial priors in several different ways. Mesh-bound approaches, such as GaussianAvatars, directly associate Gaussian primitives with a deformable facial mesh, whereas implicit deformation approaches predict continuous offsets using neural deformation fields. Other methods introduce local distance-preservation or as-isometric-as-possible constraints to improve deformation stability. IA-3DGS does not claim that the individual use of FLAME priors, Jacobian regularization, or feature memory is entirely new. Instead, its contribution lies in their coordinated integration for long-sequence monocular facial animation: semantic facial labels are retained to control region-dependent rigidity, the Jacobian constraint is applied to the complete deformation mapping rather than uniformly to all facial tissues, and periodic reference-based correction is introduced to reduce long-range identity drift. This combination is specifically evaluated under continuous sequences extending beyond 5000 frames.

## 3. Methodology

### 3.1. Overall Architecture

Given a monocular facial video sequence ${\left\{I_{t}\right\}}_{t=1}^{T}$, IA-3DGS reconstructs a subject-specific dynamic Gaussian representation in four stages. First, a 3DMM is fitted to the input video to obtain a canonical facial mesh, frame-wise head poses, and expression parameters. Gaussian primitives are initialized around the fitted facial surface and assigned anatomical region labels according to the 3DMM topology. Second, a non-rigid deformation network predicts expression-conditioned displacements in canonical space, while global head rotation and translation are explicitly applied to transform the deformed primitives into observation space. Third, a region-weighted Jacobian rigidity regularizer constrains the local deformation mapping according to the anatomical properties of each facial region. Fourth, during long-sequence inference, a cyclic memory correction mechanism periodically compares the current identity representation with a frozen reference representation and applies a smoothed correction when identity deviation is detected. The resulting Gaussian primitives are projected and alpha-composited using differentiable Gaussian rasterization. The Gaussian attributes and deformation network are jointly optimized using photometric, structural, depth, rigidity, and identity-preservation objectives.

### 3.2. 3DMM-Guided Semantic Initialization

Let the fitted facial mesh in canonical space be represented as $M=\left(V,F\right)$, where $V={\left\{v_{j}\in R^{3}\right\}}_{j=1}^{N_{v}}$ denotes the set of mesh vertices and F defines the triangular connectivity. The i-th Gaussian primitive is represented as $G_{i}=\left(\mu_{i},q_{i},s_{i},\alpha_{i},c_{i}\right)$, where $\mu_{i}\in R^{3}$ is its center, $q_{i}$ is a unit quaternion representing orientation, $s_{i}\in R_{+}^{3}$ is the anisotropic scale vector, $\alpha_{i}\in \left[0,1\right]$ is the opacity, and $c_{i}$ contains the spherical-harmonic appearance coefficients. For each Gaussian primitive, we assign an anatomical anchor $a_{i}$ on the fitted 3DMM surface and an associated semantic region label $r_{i}\in \left\{R_{qr},R_{tr},R_{exp}\right\}$. The canonical center is initialized as

(1) $$\mu_{i}^{can}=\gamma a_{i}+\left(1-\gamma \right)u_{i},\gamma \in \left[0,1\right]$$

Here, $u_{i}$ is a point sampled from the local surface patch associated with $a_{i}$, and $\gamma \in \left[0,1\right]$ balances direct anatomical anchoring and local surface coverage. Each Gaussian primitive inherits the semantic label of its associated 3DMM surface region. These labels are subsequently used to distinguish quasi-rigid, transition, and expression-sensitive regions in the region-weighted Jacobian regularization. Semantic initialization establishes anatomical correspondence but does not impose a hard positional constraint at every frame; all Gaussian attributes remain optimizable through the reconstruction objective and learned deformation field.

### 3.3. Dynamic Deformation and Region-Weighted Jacobian Regularization

For frame t, let $c_{t}$ denote the expression and local jaw-pose condition obtained from the fitted 3DMM. The non-rigid deformation network $\Phi_{\theta }$ predicts the canonical-space displacement of the i-th Gaussian center:

(2) $$\Delta \mu_{i}^{t}=\Phi_{\theta }\left(\mu_{i}^{can};c_{t}\right),\;\;\mu_{i}^{nr}\left(t\right)=\mu_{i}^{can}+\Delta \mu_{i}^{t}$$

The global head pose is represented by a rotation matrix $R_{t}\in SO\left(3\right)$ and a translation vector $T_{t}\in R^{3}$. The observation-space center is therefore computed as

(3) $$\mu_{i}^{obs}\left(t\right)=R_{t}\mu_{i}^{nr}\left(t\right)+T_{t}$$

This explicit decomposition prevents the non-rigid deformation network from absorbing global rigid head motion and reduces ambiguity between local facial deformation and head pose. Since implicit MLPs lack endogenous constraints based on physical laws, the accumulation of minute prediction residuals over thousands of frames can cause non-physical stretching of the Gaussian point cloud (i.e., the “jelly effect”). To formalize long-horizon identity drift, we distinguish the feed-forward deformation prediction from the temporally maintained identity state. The deformation MLP predicts the displacement of each Gaussian independently from its canonical position and the current expression-and-pose condition. Let $h_{t}^{*}$ and ${\hat{h}}_{t}$ denote the ideal and estimated identity states, respectively, maintained by the cyclic memory pathway after rendering and identity-feature extraction.

$${\hat{h}}_{t}=F_{\theta }\left({\hat{h}}_{t-1},c_{t}\right),\;\;h_{t}^{*}=F^{*}\left(h_{t-1}^{*},c_{t}\right),\;\;e_{t}={\hat{h}}_{t}-h_{t}^{*}$$

A first-order expansion around the ideal state gives

(4) $$e_{t}\approx A_{t}e_{t-1}+\varepsilon_{t},\;\;A_{t}={\left.\frac{\partial F_{\theta }\left(h,c_{t}\right)}{\partial h}\right|}_{h=h_{t-1}^{*}}$$

Here, $A_{t}$ is the temporal state-transition Jacobian, and $\varepsilon_{t}$ represents the combined frame-wise residual caused by imperfect deformation prediction, 3DMM tracking, rendering, and identity-feature extraction. Recursively expanding Equation (4) yields

(5) $$e_{T}\approx \left(\prod_{k=1}^{T}A_{k}\right)e_{0}+\sum_{i=1}^{T}\left(\prod_{k=i+1}^{T}A_{k}\right)\varepsilon_{i}$$

If $∥A_{t}{∥}_{2}\leq \rho$ and $∥\varepsilon_{t}{∥}_{2}\leq \varepsilon$, the accumulated error is bounded by

(6) $${∥e_{T}∥}_{2}\leq \rho^{T}{∥e_{0}∥}_{2}+\varepsilon \sum_{i=0}^{T-1}\rho^{i\;\;}$$

When ρ≈1, this bound grows approximately linearly; when ρ>1, earlier residuals may be amplified by subsequent state transitions. Therefore, even small frame-wise MLP residuals can produce observable geometric and identity drift over thousands of frames. The cyclic memory correction module reduces this growth by periodically aligning the current state with the frozen reference representation. In particular, a correction is applied every K frames and acts as a contraction satisfying

$${∥e_{mK}^{+}∥}_{2}\leq \beta {∥e_{mK}^{-}∥}_{2},\;\;0<\beta <1.$$

The correction therefore reduces the magnitude of the accumulated error at each correction step rather than allowing it to propagate without control. Because facial motion contains locally quasi-rigid regions, including the forehead, nasal bridge, and parts of the mandibular surface, we introduce a region-dependent Jacobian regularizer into the deformation field. For the deformation mapping, the local geometric transformation at a sampled canonical point p is described by the Jacobian matrix in Equation (7).

(7) $$J=\left[\begin{array}{ccc} \frac{\partial \Phi_{x}}{\partial x} & \frac{\partial \Phi_{x}}{\partial y} & \frac{\partial \Phi_{x}}{\partial z}\\ \frac{\partial \Phi_{y}}{\partial x} & \frac{\partial \Phi_{y}}{\partial y} & \frac{\partial \Phi_{y}}{\partial z}\\ \frac{\partial \Phi_{z}}{\partial x} & \frac{\partial \Phi_{z}}{\partial y} & \frac{\partial \Phi_{z}}{\partial z} \end{array}\right]$$

A locally rigid transformation has an orthogonal Jacobian satisfying $J_{p}^{T}J_{p}=I_{3}$. Based on this physical prior, the corresponding unweighted rigidity penalty is

(8) $$L_{rigid}=\sum_{p\in S}{\left\|J_{p}^{T}J_{p}-I\right\|}_{F}^{2}$$

It should be emphasized that the Jacobian rigidity term is not intended to model the entire face as a globally rigid object. Facial motion contains both approximately rigid components, such as the cranium, forehead, nasal bridge, and local mandibular surface, and highly non-rigid components, such as the lips, cheeks, eyelids, and nasolabial regions. We therefore use the semantic labels inherited from the 3DMM topology to divide the facial Gaussian primitives into a quasi-rigid region $R_{qr}$, an expression-sensitive region $R_{exp}$, and a transition region $R_{tr}$. The actual training objective therefore uses the following region-weighted form:

(9) $$L_{rigid}=\frac{1}{\sum_{p\in S}w_{p}}\sum_{p\in S}w_{p}{∥J_{p}^{T}J_{p}-I_{3}∥}_{F\;}^{2}$$

Here, $w_{p}$ is determined by the semantic region containing the sampled point p. We assign a relatively large weight to $R_{qr}$, an intermediate weight to $R_{tr}$, and a smaller but nonzero weight to $R_{exp}$, such that $w_{qr}>w_{tr}>w_{exp}>0$. The weights are smoothly interpolated near semantic boundaries to avoid discontinuities in the deformation field. Consequently, the constraint strongly suppresses non-physical shear and anisotropic stretching in anatomically stable regions while retaining sufficient degrees of freedom for muscle contraction, lip opening, cheek expansion, and eyelid motion. The global coefficient $\lambda_{rigid}$ controls the overall contribution of this term, whereas the regional weights determine its local strength. The photometric, structural-similarity, depth, and identity losses further counterbalance the rigidity term in expression-sensitive regions. Thus, the proposed regularization encourages local near-isometry only where it is anatomically appropriate, rather than imposing a hard rigid-body assumption on all facial tissues.

### 3.4. Cyclic Memory Correction Mechanism

During initialization, the system extracts and freezes an identity-sensitive reference feature from the reference frame. During long-sequence inference, the identity feature of the current rendered frame is evaluated every K = 50 frames. The cosine-distance deviation score is defined as

(10) $$d_{t}=1-\frac{{\left(f_{t}^{cur}\right)}^{T}f_{ref}}{{∥f_{t}^{cur}∥}_{2}{∥f_{ref}∥}_{2}}$$

When $d_{t}$ exceeds a predefined threshold δ, the current identity representation is aligned with the frozen reference to obtain a corrected feature $f_{t}^{corr}$.

(11) $$f_{t}=\alpha f_{t}^{corr}+\left(1-\alpha \right)f_{t-1},\;\;\alpha \in (0,1]\;$$

Here, α controls the correction strength. Periodic identity-feature evaluation limits accumulated identity drift, whereas exponential moving average smoothing reduces abrupt changes at correction positions.

### 3.5. Joint Training Objective

The subject-specific IA-3DGS model is optimized using five complementary objectives: photometric reconstruction, structural similarity, relative depth, Jacobian rigidity, and identity preservation.

(12) $$L_{total}=\lambda_{color}L_{color}+\lambda_{ssim}L_{ssim}+\lambda_{depth}L_{depth}+\lambda_{rigid}L_{rigid}+\lambda_{id}L_{id}$$

The five coefficients in Equation (12) denote the outer weights assigned to the photometric, structural-similarity, relative-depth, Jacobian-rigidity, and identity-preservation losses, respectively. The mixed photometric loss preserves pixel-level color and appearance consistency:

(13) $$L_{color}=\left(1-\lambda_{mse}\right){∥{\hat{I}}_{t}-I_{t}^{gt}∥}_{1}+\lambda_{mse}{∥{\hat{I}}_{t}-I_{t}^{gt}∥}_{2}^{2}$$

Here, ${\hat{I}}_{t}$ and $I_{t}^{gt}$ denote the rendered and ground-truth images, respectively. The internal mixing coefficient $\lambda_{mse}\in \left[0,1\right]$ balances the $L_{1}$ and squared $L_{2}$ components and is distinct from the outer photometric-loss weight in Equation (12). The structural-similarity loss preserves local luminance, contrast, and structural information:

(14) $$L_{ssim}=1-SSIM\left({\hat{I}}_{t},I_{t}^{gt}\right)$$

The SSIM operator compares the rendered image with the corresponding ground-truth image. A lower value of this loss indicates greater structural similarity. The scale-and-shift-normalized relative-depth loss reduces front-to-back depth inconsistency:

(15) $$L_{depth}=\frac{1}{\left|\Omega_{t}\right|}\sum_{u\in \Omega_{t}}\left|{\tilde{D}}_{t}^{render}\left(u\right)-{\tilde{D}}_{t}^{3DMM}\left(u\right)\right|$$

In Equation (15), $\Omega_{t}$ is the valid foreground region, ${\tilde{D}}_{t}^{render}$ is the normalized depth map rendered from the Gaussian representation, and ${\tilde{D}}_{t}^{3DMM}$ is the normalized depth map rasterized from the fitted 3DMM. Scale-and-shift normalization makes the loss sensitive to relative facial geometry rather than to an arbitrary monocular depth scale. The identity-preservation loss uses a frozen pretrained face-recognition network $f_{id}$. The normalized embeddings and their cosine-distance loss are defined as

(16) $$\begin{array}{rr} {\hat{v}}_{t} & =normalize\left[f_{id}\left({\hat{I}}_{t}\right)\right],\\ v_{t}^{gt} & =normalize\left[f_{id}\left(I_{t}^{gt}\right)\right]\\ L_{id} & =1-{\hat{v}}_{t}^{T}v_{t}^{gt}. \end{array}$$

**Figure 2:**
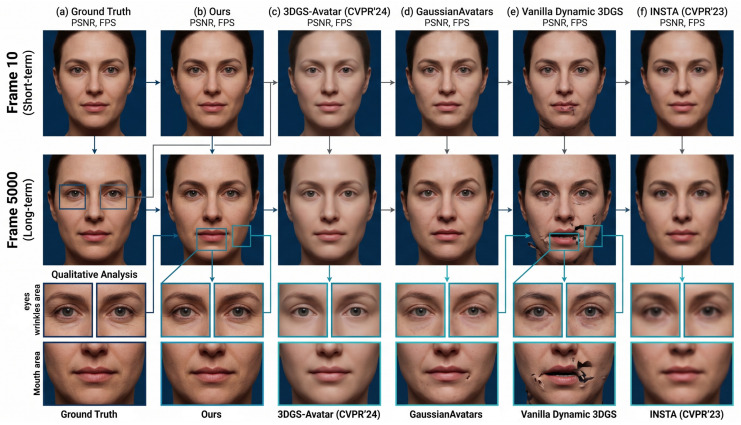
Qualitative comparison of facial rendering at an early frame and a long-sequence frame. All enlarged eye and mouth regions use identical crop coordinates and display scales.

The cosine-distance loss penalizes changes in identity-sensitive embedding direction. The region-weighted Jacobian rigidity loss is defined in Equation (9). The outer weights for the photometric, structural-similarity, relative-depth, Jacobian-rigidity, and identity-preservation losses were set to 1.0, 0.2, 0.5, 10.0, and 1.0, respectively. The loss functions and their corresponding weights are summarized in Table 1. A qualitative comparison of facial rendering at an early frame and a long-sequence frame is shown in Figure 2.

## 4. Experiments and Analysis of Results

To evaluate the rendering fidelity and long-term temporal stability of IA-3DGS, we conducted experiments on multiple facial video datasets and compared the method with representative dynamic neural rendering baselines through quantitative, qualitative, and cumulative controlled component analyses.

### 4.1. Experimental Setup

We evaluated IA-3DGS on two public facial-avatar datasets and one self-collected long-sequence dataset:

1. NeRSemble dataset: Contains ultra-high-resolution (>4K) dynamic facial video sequences. We selected long sequence clips of 5 different subjects from this dataset and used only single-view (frontal view) images to simulate a monocular input scenario in order to test the model’s ability to reconstruct high-frequency details under extreme micro-expressions.
2. NHA (Neural Head Avatars) dataset: A benchmark dataset designed specifically for monocular head reconstruction, featuring a wide range of head poses and complex facial expressions, used to evaluate the robustness of deformation fields under vigorous motion.
3. Custom-5K: Dataset Acquisition and Partitioning. To evaluate cumulative degradation over continuous monocular facial sequences, we collected a Custom-5K dataset containing speech and facial-expression sequences from three participants with different facial geometries and appearances. Each sequence was recorded under controlled lighting at a resolution of 1920 × 1080 pixels and contains more than 5000 consecutive frames. The data-collection protocol was approved by the relevant Institutional Review Board, and written informed consent was obtained from all participants. Each identity was modeled using an independent subject-specific model. The first 500 consecutive frames of each sequence were used for training, the following 100 frames were used for validation, and all remaining frames were reserved for continuous long-sequence testing. FLAME Tracker and DECA were used to estimate frame-wise camera poses, facial-expression parameters, and head-pose parameters. No test frame was used during model training or validation.

### 4.2. Evaluation Metrics

Standard reconstruction quality was evaluated using peak signal-to-noise ratio (PSNR), structural similarity (SSIM), and learned perceptual image patch similarity (LPIPS). Higher PSNR and SSIM values indicate better reconstruction quality, whereas lower LPIPS values indicate better perceptual similarity. Long-sequence stability was evaluated using long-term LPIPS (L-LPIPS), landmark mean error (LME), and temporal flickering error (TFE). At a selected long-sequence position T, L-LPIPS is defined as the LPIPS distance between the rendered image and its corresponding ground-truth image:

(17) $$L-LPIPS\left(T\right)=LPIPS\left({\hat{I}}_{T},I_{T}^{gt}\right)$$

The landmark mean error is defined as follows:

(18) $$LME\left(T\right)=\frac{1}{N_{l}}\sum_{j=1}^{N_{l}}{∥{\hat{l}}_{j,T}-l_{j,T}^{gt}∥}_{2},\;\;N_{l}=68$$

Here, ${\hat{l}}_{j,T}$ and $l_{j,T}^{gt}$ denote the predicted and ground-truth positions of the j-th facial landmark at frame T, respectively. LME is reported in millimeters. Lower L-LPIPS, LME, and TFE values indicate better perceptual-detail preservation, geometric consistency, and temporal stability, respectively.

### 4.3. Implementation Details and Computational Cost

All experiments were conducted on a single NVIDIA RTX 4090 (24 GB VRAM) using the PyTorch 1.12.1 framework. The maximum number of active Gaussian primitives was limited to 1,000,000. We used the Adam optimizer, with learning rates dynamically decaying for the 3D Gaussian attributes and the MLP deformation field, respectively. The Jacobian rigidity coefficient was set to 10.0, and cyclic memory correction was evaluated every 50 frames. The total number of training iterations for the model was 40,000 (taking approximately 45 min). During the inference phase, thanks to the efficiency of the underlying CUDA rasterization, the system was able to achieve a real-time rendering frame rate of approximately 48 FPS at 1080p resolution, even with the deformation network included. Regarding the network architecture, the implicit non-rigid deformation field $\Phi_{\theta }$ is parameterized by a six-layer multilayer perceptron (MLP) with a hidden dimension of 256 and ReLU activations, ensuring sufficient capacity to model high-frequency facial muscle dynamics while maintaining real-time inference efficiency.

The identity-preservation components have different computational profiles. The 3DMM-guided semantic initialization is performed once before joint optimization and therefore does not add steady-state rendering cost. The Jacobian rigidity term requires automatic differentiation at the sampled canonical points during training, but it is not evaluated during inference and does not alter the inference graph. Cyclic memory correction performs identity-feature evaluation only once every K=50  frames; the remaining frames use the standard deformation and Gaussian-rasterization path. The reported throughput of approximately 48 FPS at 1920×1080  represents the end-to-end speed of the complete IA-3DGS framework. Because no module-isolated timing was recorded, we do not report an unsupported numerical overhead for the individual modules. The 35 FPS value reported for Vanilla Dynamic 3DGS in Table 2 is an overall baseline throughput and should not be interpreted as a module-level estimate of the proposed components’ overhead.

### 4.4. Baselines

We compared IA-3DGS with the current state-of-the-art (SOTA) models in the field of 3D dynamic head reconstruction:

1. INSTA (CVPR 2023) [14]: A NeRF-based head-avatar method conditioned on 3DMM priors.
2. Vanilla Dynamic 3DGS [11]: A dynamic 3DGS baseline based on an implicit MLP deformation field, without semantic anchors, Jacobian rigidity constraints, or cyclic memory correction.
3. GaussianAvatars (CVPR 2024) [5]: A FLAME-driven head-avatar method that attaches 3D Gaussian primitives to a parametric facial mesh.
4. 3DGS-Avatar (CVPR 2024) [4]: An animatable-avatar method that combines deformable 3D Gaussian primitives with an MLP-based deformation field.

### 4.5. Quantitative Assessment

#### 4.5.1. Reconstruction Quality on Standard-Length Sequences

We first evaluated standard image synthesis quality on the NeRSemble and NHA datasets (clip lengths of 300–500 frames). As shown in Table 2, the evaluated 3DGS-based methods achieved higher reconstruction metrics and rendering speeds than INSTA under the reported experimental settings. Among the evaluated methods, IA-3DGS obtained the highest numerical PSNR and SSIM values and the lowest numerical LPIPS value while retaining real-time rendering. These results indicate that the combined reconstruction objective and 3DMM-guided dense surface initialization preserve local facial appearance under the evaluated standard-length conditions.

#### 4.5.2. Long-Term Temporal Stability

We evaluated cumulative identity and geometric degradation on the Custom-5K long-sequence dataset. Table 3 reports L-LPIPS and LME at frames 1000 and 5000. Table 3 shows that the differences between the evaluated methods become more pronounced as the sequence length increases. At frame 5000, IA-3DGS obtained an L-LPIPS of 0.046 and an LME of 1.3 mm. In comparison, Vanilla Dynamic 3DGS obtained an L-LPIPS of 0.245 and an LME of 6.8 mm, whereas 3DGS-Avatar obtained values of 0.118 and 3.5 mm, respectively. These results indicate that the proposed semantic anchoring, region-weighted deformation regularization, and cyclic correction reduce cumulative perceptual and geometric degradation under the evaluated long-sequence conditions.

#### 4.5.3. Qualitative Assessment

For the qualitative assessment of visual quality, we extracted close-up renderings from frames 3000 and 5000 of the long-duration sequence for comparison.

**Figure 3:**
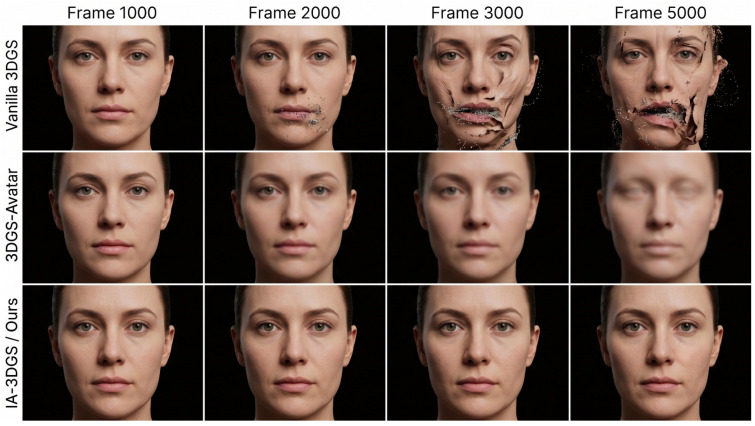
Qualitative comparison of long-sequence facial rendering at frames 1000, 2000, 3000, and 5000.

Figure 3 illustrates the temporal evolution of the rendered results. Vanilla Dynamic 3DGS develops progressively stronger geometric artifacts around the mouth in the later portion of the sequence. 3DGS-Avatar maintains the overall facial contour but exhibits increasing local texture smoothing. In contrast, IA-3DGS produces comparatively stable facial contours and local appearance throughout the displayed sequence. These visual observations are consistent with the L-LPIPS and LME results reported in Table 3.

**Figure 4:**
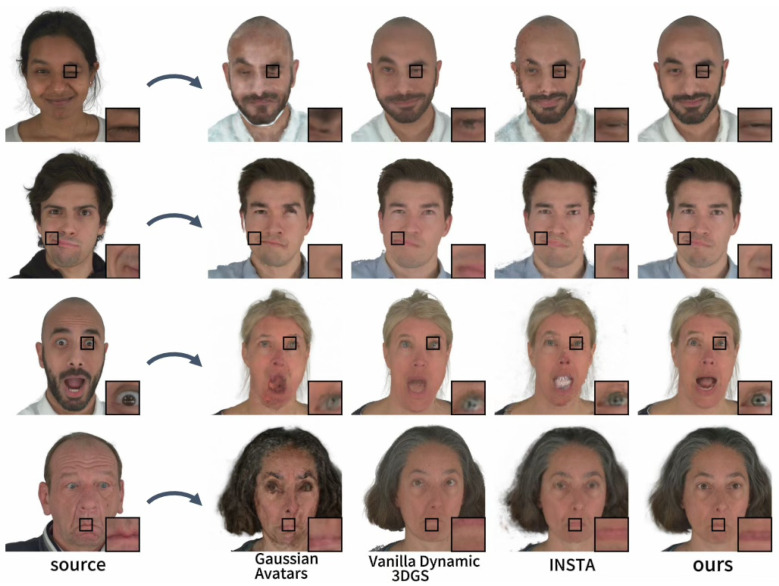
Qualitative comparison in cross-identity facial reenactment. The source subject provides the expression and pose sequence, whereas each method reconstructs the appearance of the target identity.

Figure 4 presents qualitative cross-identity facial reenactment results. In this setting, the source subject provides the facial-expression and pose conditions, whereas the target sequence provides the identity-specific appearance. IA-3DGS maintains the displayed target facial characteristics while transferring the source expression. GaussianAvatars and INSTA preserve the overall facial shape but show varying degrees of local appearance smoothing, whereas Vanilla Dynamic 3DGS produces more visible artifacts in highly deformable facial regions. Because an exactly paired target ground-truth frame is unavailable for this cross-identity setting, this figure is used as a qualitative comparison rather than as evidence of pixel-level reconstruction accuracy.

### 4.6. Controlled Component Analysis

To avoid conflating the effects of multiple modules, we report the existing measurements as a cumulative controlled component analysis rather than as a leave-one-out ablation. All configurations use the same NeRSemble evaluation setting at frame 3000. Starting from the purely implicit baseline, the modules are added sequentially: 3DMM semantic anchoring, region-weighted Jacobian rigidity regularization, and cyclic memory correction. Consequently, each adjacent pair differs by exactly one added component. The configurations and corresponding quantitative results are summarized in Table 4.

The transition from configuration D to C isolates the addition of 3DMM semantic anchoring: PSNR increases from 27.65 to 29.82 dB, L-LPIPS decreases from 0.155 to 0.086, and TFE decreases from 0.042 to 0.035. The transition from C to B isolates the addition of region-weighted Jacobian rigidity regularization: PSNR increases from 29.82 to 31.05 dB, L-LPIPS decreases from 0.086 to 0.052, and TFE decreases from 0.035 to 0.018. Finally, the transition from B to A isolates cyclic memory correction: PSNR increases from 31.05 to 31.68 dB and L-LPIPS decreases from 0.052 to 0.041, whereas TFE changes slightly from 0.018 to 0.019. The small increase in TFE is consistent with minor local variation at periodic correction positions, whereas the lower L-LPIPS indicates improved long-range identity preservation. These sequential comparisons distinguish the contributions of the semantic, geometric, and temporal components without treating configuration C as a leave-one-out model that retains cyclic memory correction.

### 4.7. Limitations and Future Directions

The present evaluation has several limitations. First, every model is trained separately for one identity. The experiments therefore demonstrate subject-specific reconstruction and long-sequence stability but do not establish cross-subject generalization or the ability of a single model to represent unseen identities. Second, Custom-5K contains only three participants recorded under controlled lighting. This restricted cohort and capture environment limit conclusions about performance across broader populations, illumination conditions, backgrounds, and acquisition devices. Third, the method depends on the accuracy of 3DMM-based tracking and may degrade under severe occlusion, extreme expressions, or large unobserved head rotations. Finally, monocular input provides insufficient geometric evidence for completely unobserved facial regions, and the current spherical-harmonic appearance representation does not explicitly disentangle illumination, reflectance, and geometry.

Future work will investigate multi-subject or identity-conditioned Gaussian models that share deformation priors while retaining subject-specific anchors. Broader evaluation should include more identities, less constrained capture conditions, and cross-dataset testing. More robust semantic tracking, multi-view observations or generative geometry priors, and relightable Gaussian representations may further improve generalization to occlusion, unseen viewpoints, and changing illumination.

## 5. Conclusions

This paper presented IA-3DGS, a subject-specific dynamic Gaussian framework for long-sequence facial animation from monocular video. The method combines 3DMM-guided semantic initialization, region-weighted Jacobian rigidity regularization, and cyclic identity correction. Semantic initialization establishes anatomical correspondence for Gaussian primitives, the region-dependent Jacobian term reduces non-physical local deformation while preserving expression-sensitive motion, and periodic reference-based correction mitigates cumulative identity degradation. Experiments on NeRSemble, NHA, and Custom-5K showed that IA-3DGS achieved competitive standard-sequence reconstruction quality and improved long-sequence perceptual and geometric consistency relative to the evaluated baselines. On standard-length sequences, the method achieved a PSNR of 31.85 dB, an SSIM of 0.942, and an LPIPS of 0.038. At frame 5000 of the Custom-5K sequences, IA-3DGS obtained an L-LPIPS of 0.046 and an LME of 1.3 mm, compared with 0.245 and 6.8 mm, respectively, for the purely implicit dynamic 3DGS baseline. The framework rendered at approximately 48 FPS at 1920 × 1080 resolution on a single NVIDIA RTX 4090. Within the evaluated subject-specific monocular setting, the results support the coordinated use of semantic anchoring, local deformation regularization, and periodic identity correction for long-duration facial animation.

## Figures and Tables

**Table 1:** Loss functions and weights used for subject-specific IA-3DGS training.

Loss Term	Description	Weight	Objective
$L_{color}$	Mixed $L_{1}$ and squared $L_{2}$ photometric loss	1.0	Maintains pixel-level color and appearance consistency
$L_{ssim}$	Structural-similarity loss	0.2	Preserves local image structure
$L_{depth}$	Scale-and-shift-normalized relative-depth loss	0.5	Reduces front–back depth inconsistency
$L_{rigid}$	Region-weighted Jacobian rigidity loss	10.0	Reduces non-physical local deformation
$L_{id}$	Identity-embedding cosine-distance loss	1.0	Improves identity consistency

**Table 2:** Quantitative comparison of reconstruction quality and rendering speed on standard-length sequences.

Method	PSNR ↑	SSIM ↑	LPIPS ↓	FPS ↑
INSTA	28.15	0.892	0.085	12
Vanilla Dyn-3DGS	29.80	0.905	0.062	35
GaussianAvatars	31.12	0.932	0.045	<10
3DGS-Avatar	31.54	0.938	0.041	110
IA-3DGS (Ours)	31.85	0.942	0.038	48

**Table 3:** Long-sequence perceptual and geometric stability on the Custom-5K dataset.

Method	L-LPIPS at 1000 ↓	L-LPIPS at 5000 ↓	LME at 1000 (mm) ↓	LME at 5000 (mm) ↓
INSTA (CVPR’23)	0.075	0.155	1.6	4.2
Vanilla Dyn-3DGS	0.082	0.245	2.1	6.8
GaussianAvatars	0.045	0.058	1.1	1.8
3DGS-Avatar	0.082	0.118	1.2	3.5
IA-3DGS (Ours)	0.040	0.046	1.0	1.3

**Table 4:** Cumulative controlled component analysis of IA-3DGS at frame 3000.

Configuration	PSNR ↑	L-LPIPS ↓	TFE ↓
(A) Full IA-3DGS	31.68	0.041	0.019
(B) Semantic Anchoring + Jacobian	31.05	0.052	0.018
(C) Semantic Anchoring Only	29.82	0.086	0.035
(D) Purely Implicit Baseline	27.65	0.155	0.042

## Data Availability

Due to privacy and ethical restrictions associated with the high-resolution human facial video data used in our research, we cannot make the dataset publicly available in an open-source repository. However, the data presented in this study are available upon reasonable request. Interested researchers may contact the corresponding author via email to request access to the dataset.
